# Complementary roles of KCa3.1 channels and β1-integrin during alveolar epithelial repair

**DOI:** 10.1186/s12931-015-0263-x

**Published:** 2015-09-04

**Authors:** Alban Girault, Jasmine Chebli, Anik Privé, Nguyen Thu Ngan Trinh, Emilie Maillé, Ryszard Grygorczyk, Emmanuelle Brochiero

**Affiliations:** Centre de recherche du Centre hospitalier de l’Université de Montréal (CRCHUM), Tour Viger, 900 rue Saint-Denis, Montréal, Québec H2X0A9 Canada; Département de médecine, Université de Montréal, CP6128, Succursale Centre-ville, Montréal, Québec H3C3J7 Canada

## Abstract

**Background:**

Extensive alveolar epithelial injury and remodelling is a common feature of acute lung injury and acute respiratory distress syndrome (ARDS) and it has been established that epithelial regeneration, and secondary lung oedema resorption, is crucial for ARDS resolution. Much evidence indicates that K^+^ channels are regulating epithelial repair processes; however, involvement of the KCa3.1 channels in alveolar repair has never been investigated before.

**Results:**

Wound-healing assays demonstrated that the repair rates were increased in primary rat alveolar cell monolayers grown on a fibronectin matrix compared to non-coated supports, whereas an anti-β1-integrin antibody reduced it. KCa3.1 inhibition/silencing impaired the fibronectin-stimulated wound-healing rates, as well as cell migration and proliferation, but had no effect in the absence of coating. We then evaluated a putative relationship between KCa3.1 channel and the migratory machinery protein β1-integrin, which is activated by fibronectin. Co-immunoprecipitation and immunofluorescence experiments indicated a link between the two proteins and revealed their cellular co-distribution. In addition, we demonstrated that KCa3.1 channel and β1-integrin membrane expressions were increased on a fibronectin matrix. We also showed increased intracellular calcium concentrations as well as enhanced expression of TRPC4, a voltage-independent calcium channel belonging to the large TRP channel family, on a fibronectin matrix. Finally, wound-healing assays showed additive effects of KCa3.1 and TRPC4 inhibitors on alveolar epithelial repair.

**Conclusion:**

Taken together, our data demonstrate for the first time complementary roles of KCa3.1 and TRPC4 channels with extracellular matrix and β1-integrin in the regulation of alveolar repair processes.

## Background

Extensive damage and remodelling of the alveolar epithelium occur in various lung pathologies, including acute lung injury (ALI) and its more severe form, acute respiratory distress syndrome (ARDS) [1–3]. Alveolar regeneration, which is crucial to restore alveolar epithelial integrity and function, is thus a critical component of ARDS resolution and patient recovery [1, 2, 4].

After damage, several cellular events are engaged in an attempt to restore alveolar integrity, including changes in cell-matrix adhesion through the action of matrix metalloproteinases and integrin receptors, cytoskeleton reorganization, cell spreading and migration, as well as cell proliferation and differentiation [5]. These complex processes integrate multiple mechanisms and proteins, which are regulated by various components such as growth factors, growth factor receptors and downstream signalling pathways [5–7]. Integrins play an active role in epithelial repair, not only by creating a link between the ECM and cell cytoskeleton but also by interacting with proteins involved in cell migration and proliferation, including growth factor receptors, protein kinases as well as ion channels [8–11]. β1-integrin, for example, has been shown to regulate alveolar type II (ATII) cell migration on fibronectin matrix [12]. Moreover, increased levels of fibronectin and collagen have been detected in lung tissues from patients with ARDS [13].

Increasing evidence also indicates a function of potassium (K^+^) channels in the regulation of epithelial repair processes [14]. More precisely, silencing or inhibition of different types of K^+^ channels has been reported to decrease epithelial cell proliferation [15–18], motility [15, 16, 19–23] and differentiation [20], as well as epithelial wound repair [15, 16, 24–26]. Our data on primary rat ATII cells previously highlighted an involvement of two types of K^+^ channels, i.e. KvLQT1 and K_ATP_, in the control of cell proliferation, motility and repair [15]. A role for KCa3.1 channels in airway ion transport [27, 28], as well as repair processes of several epithelial tissues [16, 22, 29] has also been established; however, the contribution of this channel in alveolar repair has not been explored before.

The mechanisms whereby K^+^ channels control epithelial repair processes may be multiple, including changes in membrane potential, cell volume and shape, [Ca^2+^]_i_ and various signalling pathways (for review see [14]). In addition, several reports indicated that different types of K^+^ channels (e.g. BKCa, Kv1.3, hERG, GIRK, Kir4.2) could also directly interact with migratory machinery proteins, such as β1-integrins [30–33]. However, to the best of our knowledge, a relationship between the KCa3.1 and β1-integrin in epithelial cells has never been investigated before.

Based on these data, we postulated that KCa3.1 and β1-integrin play a complementary role during alveolar epithelial repair. We thus evaluated the roles of extracellular fibronectin matrix, β1-integrin and KCa3.1 channels in alveolar repair processes, especially cell migration, proliferation and wound healing after mechanical injury. Finally, the regulation and complementary function of TRPC4 Ca^2+^ channels were explored.

## Methods

### Alveolar epithelial type II cell isolation and primary culture

Alveolar epithelial cells were isolated from rat lungs according to a procedure approved by our institutional animal care committee (CIPA) of Centre de Recherche du Centre Hospitalier de l’Université de Montréal (CRCHUM) in accordance with the Canadian Council of Animal Care (CCAC) standards. Alveolar epithelial type II (ATII) cells were isolated from adult male Sprague–Dawley rats (6–7 weeks), according to a well-established protocol [15, 34–36]. In brief, the lungs were washed to remove blood cells and alveolar macrophages before treatment with elastase (Worthington, Lakewood, NJ, USA). They were then minced, and the resulting suspension was filtered. Alveolar cells were collected and purified using a differential adherence technique [37], which enhances the purity of the ATII cell pool up to 86 % [35, 38]. Although most of the macrophages are bound on IgG coated plates during this differential adherence technique, some retained in the final post-IgG cell mix (along with some red blood cells), where they likely constituted a significant proportion of non-alveolar cells in the cell prep at day 0 [38]. This freshly isolated cell suspension was seeded on Petri dishes (Corning, Fisher Scientific Ltd., Nepean, ON, Canada) or on glass slides (VWR International, Mississauga, ON, Canada) and adherent alveolar epithelial cells were cultured in minimal essential medium (MEM; Gibco, Life Technologies Inc., Burlington, ON, Canada) containing 10 % FBS (Gibco, Life Technologies Inc.), 0.08 mg/l gentamicin, septra (3 μg/ml trimethoprime + 17 μg/ml sulfamethoxazole), 0.2 % NaHCO_3_ (Sigma-Aldrich, Oakville, ON, Canada), 10 mM HEPES (Hyclone, Fisher Scientific Ltd.), and 2 mM L-glutamine (Gibco, Life Technologies Inc.), as previously described [15, 35]. The MEM-FBS-septra medium was replaced after 3 days by the same MEM-FBS without septra.

### Wound-healing assay

ATII cells, cultured for 3–4 days, were injured mechanically with a pipette tip (six wounds per Petri dish) according to a highly reproducible technique [15, 16, 39–42]. This commonly employed “wound-healing assay” allows for the study of early mechanisms engaged after injury, i.e. cell migration and proliferation [39]. A mark on the Petri dishes enabled us to photograph the wounds at exactly the same place at various times (at time 0 after injury and after 24 h of repair). The rate of wound closure, presented in μm^2^/h, was calculated with ImageJ software (National Institutes of Health, Bethesda, MD, USA) from the wound area measured after repair compared with the initial wound area, for each wound.

### Proliferation assay

ATII cells were seeded at low density (52,000/cm^2^) in 35-mm Petri dishes for 3 days in MEM-FBS-septra medium and then exposed or not to TRAM-34 (Sigma-Aldrich) for a 24-h period. Cell growth was evaluated by counting the number of ATII cells by separation with trypsin-EDTA (0.05 %, Gibco, Life Technologies Inc.) before (at day 3, T0) and after (at day 4, T24h) treatment. The absence of drug cytotoxicity was verified by trypan-blue exclusion assay. The number of ATII cells was also counted at day 3 after transfection with negative control or KCa3.1 siRNAs (see section on siRNA transfection).

### Cell migration assays

ATII cell migration was first evaluated by Boyden-type chamber assays, as previously described [15, 39]. Briefly, primary ATII cells were separated with trypsin-EDTA (Gibco, Life Technologies Inc.), counted, and their viability was verified with Trypan blue assay (Sigma-Aldrich). A series of experiments was also performed on ATII cells previously transfected with negative control or KCa3.1 siRNAs (see section on siRNA transfection). The cell suspensions (75,000 cells in FBS-free MEM) were placed in the upper compartment of 8-μm pore filters (0.33 cm^2^, ThinCerts-TC inserts, Greiner Bio-one; MJS Biolynx, Brockville, ON, Canada) coated on the lower side with a gelatin (control condition, Sigma-Aldrich) or fibronectin matrix (Sigma-Aldrich). The lower compartment was filled with FBS-free MEM in the absence or presence of TRAM-34. After an 18-h migration period, the filters were washed with PBS, the cells were fixed with paraformaldehyde-acetone solution and then stained with hematoxylin (Sigma-Aldrich). Non-migrating cells in the upper compartment were scraped off with cotton-tipped applicators (Fisher Scientific Ltd.), whereas migrating cells on the lower face of the filters were counted in different randomly chosen fields under a light-inverted microscope at x20 magnification. Two-D cell migration rates (μm/h) of single cells were also evaluated by single-cell tracking in live-video microscopy experiments. Images were captured at 5-min intervals over a 24-h period by digital camera connected to Zeiss microscope. The migration rates and cell trajectories were analyzed by AxioVision software (Carl Zeiss, Jena, Germany).

### Immunoblotting of enriched ATII membrane fractions and co-immunoprecipitation of KCa3.1 and ß1-integrin

For enriched membrane fraction assays, ATII cells were scraped into TRIS-sucrose buffer (20 mM TRIS HCl (Life Technologies Inc.), 5 mM EDTA (ACP, Montréal, QC, Canada), 200 mM sucrose (Sigma-Aldrich), protease inhibitor cocktail (Complete Mini EDTA-free protease inhibitor cocktail, Roche Applied Science, Laval, QC, Canada)) and then homogenized with a glass putter for 1 min. Cell lysates were centrifuged 15 min at 3,000 rpm (4 °C), supernatants were collected and then centrifuged at 38,000 rpm for 1 h30 at 4 °C. Supernatants were harvested and enriched membrane fractions in pellet were resuspended in lysis buffer (150 mM NaCl (ACP), 50 mM Tris–HCl, pH 7.6, 1 % Triton X-100 (Fisher Scientific Ltd.), 0.1 % SDS (Bioshop Canada Inc., Burlington, ON, Canada), protease inhibitor cocktail). Protein concentrations were then measured by Bradford assay and immunoblotting was conducted as described below.

For co-immunoprecipitation assays, ATII cell lysis was performed in buffer 1 (150 mM NaCl, 50 mM Tris–HCl, pH 7.5, 1 % Nonidet P40, and 0.5 % sodium deoxycholate) from the immunoprecipitation kit (protein A, Roche Applied Science) following the manufacturer’s instructions. The lysates were homogenized and isolated by centrifugation. After quantification using the Bradford method, 1–2 mg proteins from the soluble lysate were precleared with 50 μl of 50 % protein A-agarose suspension. The precleared soluble lysates were then incubated for 1–2 h with a rabbit anti-KCa3.1 antibody (APC-064, Alomone Labs, Jerusalem, Israel) [43] or a mouse anti-β1-integrin antibody (610467, BD Biosciences, Mississauga, ON, Canada). The immunocomplexes were precipitated by overnight incubation at 4 °C with 50 μl of 50 % protein A-agarose suspension. After being washed twice with both buffer 1 (see composition above) and buffer 2 (500 mM NaCl, 50 mM Tris–HCl, pH 7.5, 0.1 % Nonidet P40, 0.05 % sodium deoxycholate) and then once with buffer 3 (10 mM Tris · HCl, pH 7.5, 0.1 % Nonidet P40, 0.05 % sodium deoxycholate), proteins bound to beads were collected by centrifugation and eluted by 30–50 μl of 2X sample buffer (62.5 mM Tris–HCl, pH 6.8, 2 % SDS, 10 % glycerol, 0.2 % bromophenol blue, 4 % β-mercaptoethanol). Control assays, in the absence of lysate or antibodies during IP, have also been performed.

Enriched membrane fraction proteins and immunoprecipitated proteins were then separated by SDS-PAGE and transferred onto nitrocellulose membranes (GE Healthcare, Mississauga, ON, Canada) according to a well-established laboratory protocol [15, 36]. After blocking, the membranes were incubated with anti-KCa3.1 (APC-064, Alomone Labs), anti-β1-integrin (610467, BD Biosciences) or anti-TRPC4 (ACC-018, Alomone Labs) antibodies. After washing, the membranes were incubated with goat anti-rabbit (for KCa3.1 and TRPC4, Cell Signaling Technology, Danvers, MA, USA) and goat anti-mouse (for β1-integrin, Millipore, Etobicoke, ON, Canada) IgG linked to horseradish peroxidase for 1 h. The intensity of each specific band was quantified with ChemiDoc XRS+ Molecular Imager and Image Lab software (Bio-Rad, Mississauga, ON, Canada).

### Immunofluorescence assay

ATII cells were seeded at low confluency on glass coverslips. On day 3, cells were fixed in 4 % paraformaldehyde (Electron Microscopy Sciences, Hatfield, PA, USA) in PBS for 20 min and permeabilized with 0.1 % triton X100 in PBS for 10 min at room temperature. After blocking in PBS containing 5 % bovine serum albumin (Fisher Scientific Ltd.) for 1 h30, cells were incubated with specific anti-KCa3.1 and β1-integrin antibodies for 1 h30. Absence of background signal and non-specific staining was verified in control experiments, by omitting primary antibodies. Cells were then incubated with anti-rabbit-CF633 (Sigma-Aldrich, for KCa3.1 detection) or Alexa Fluor 488-conjugated anti-mouse (Life Technologies Inc., for β1-integrin detection) antibodies for 45 min. Slides were finally rinsed and counterstained with DAPI (Sigma-Aldrich). Fluorescent images were captured with an ExiAqua camera (QImaging, Surrey, BC, Canada) connected to an Olympus fluorescence microscope (Olympus, Richmond Hill, ON, Canada).

### Small interfering (si)RNA transfections

After isolation, ATII cells in suspension were transfected with a combination of Lipofectamine™ RNAiMAX (Invitrogen) and Universal negative control or KCa3.1 siRNAs (Sigma-Aldrich). Five h after seeding, fresh complete MEM was added onto cells, which were then primary cultured for 3 to 4 days. Efficient transfection (>80 %) was observed in these conditions and KCa3.1 silencing (>70 %) was verified by PCR (see below).

### PCR amplifications

Five μg of total RNA purified from ATII cells with TRIzol reagent (Life Technologies Inc.) was reverse-transcribed into cDNA with MMLV reverse transcriptase (RT, Life Technologies Inc.) in the presence of oligodT primers (Invitrogen, Life Technologies Inc.). cDNAs were amplified with Taq polymerase (Life Technologies Inc.) and specific primers designed from sequences of rat KCa3.1 and TRPC channels: KCa3.1 5′-gctgttcatgactgacaacg-3′ and 5′-catagccaatggtcaggaac-3′, 500-bp product; TRPC1 5′- gattttgggaaatttctaggaatg-3′ and 5′-ctcatgatttgctatcagctgg-3′, 363-bp product; TRPC2 5′- cgttccagtttctcttctggaccat-3′ and 5′- agcatcgtcctcgatcttctgg-3′, 191-bp product; TRPC3 5′- tgatgaggtgaacgaaggtgaactg-3′ and 5′- tgcccacatttgtgccagagtca-3′, 206-bp product; TRPC4 5′- tctgcagatatctctgggaaggatgc-3′ and 5′- aagctttgttcgagcaaatttccattc-3′, 415-bp product; TRPC5 5′- cccggcatgaattcacggag-3′ and 5′- catggtcggcaatgagctggtag-3′, 129-bp product; TRPC6 5′- gagaacataggctatgttctgtatggagtc-3′ and 5′- gccatcatcctcaatttcctgg-3′, 114-bp product; TRPC7 5′- tccctttaacctggtgccgagtc-3′ and 5′- ttcagcatgcccatttccagg-3′, 129-bp product. Primer pairs were designed in distinct exons to avoid genomic DNA amplification. RT-PCR amplification was undertaken according to a well-established laboratory protocol [35, 36, 44]. PCR products were normalized with the β-actin signal for each cDNA sample (β-actin 5′-ctaaggccaaccgtgaaaag-3′ and 5′- gccatctcttgctcgaagtc-3′, 311-bp product). The RT-PCR products were finally separated on agarose gels, stained with SYBR Safe (Life Technologies Inc.). Signals were detected by Typhoon Gel Imager and analyzed by ImageQuant software (Molecular Dynamics, Baie d’Urfé, QC, Canada) [35, 36, 44].

### Fura-2 calcium imaging

Intracellular calcium concentration ([Ca^2+^]_i_) was monitored using the fluorescent dye Fura-2. Briefly, ATII cells cultured on coverslips were loaded (1 h15 at 37 °C) with 5 μM Fura-2-AM (Life Technologies Inc.) in MEM containing 0.04 % pluronic F127 (Life technologies Inc.) followed by a 45 min de-esterification in MEM. Coverslips with loaded cells were then mounted in the imaging chamber on the stage of an inverted microscope (Nikon TE300, Mississauga, ON, Canada) and bathed with a physiological saline solution (PSS, 140 mM NaCl, 5 mM KCl, 1 mM MgCl_2_, 2 mM CaCl_2_, 11.1 mM glucose, 10 mM HEPES, pH 7.4). To measure the Ca^2+^ permeability through ATII cell plasma membranes, PSS was replaced by the same solution without Ca^2+^ and supplemented with 1 mM EGTA (PSS 0 Ca^2+^). Cells were then alternately excited at 340 and 380 nm with a high-pressure mercury lamp via interference filters (Chroma Technology, Brattleboro, VT, USA) mounted on a filter wheel (Sutter Lamba 10-C, Sutter Instrument, Novato, CA, USA) with a dichroic mirror (510/540 nm, Chroma Technology). Fluorescence images were recorded at 5-s intervals by digital camera using Metafluor software (Molecular Devices, Guelph, ON, Canada) and analyzed with Metafluor Analysis (Molecular Devices). Fura-2 fluorescence measurements (corresponding to the mean fluorescence values of 30 cells/field) are presented as [Ca^2+^]_i_, which was calculated accordingly to the Grynkiewicz equation [45], using a Kd of 225 nM. Rmax was determined by the addition of digitonin (4 μM, Sigma-Aldrich) to PSS (2 mM Ca^2+^) and Rmin by subsequent addition of ethylene glycol-bis ([β-amino-ethyl ether)-N,N,N’,N’-tetraacetic acid (EGTA) at a final concentration of 20 mM.

### Statistical analysis

The data are presented as mean ± standard error of the mean (SEM). Statistical analyses were performed with GraphPad Prism version 5 for Windows (GraphPad software, San Diego, CA, USA) using Wilcoxon signed rank test and one sample sign test. Two-way Anova tests were performed with IBM SPSS Statistics 22 (IBM Corp., Armonk, NY). Differences were considered significant when *p* < 0.05.

## Results

### Involvement of KCa3.1 in ATII epithelial repair on fibronectin matrix

**Fig. 1:**
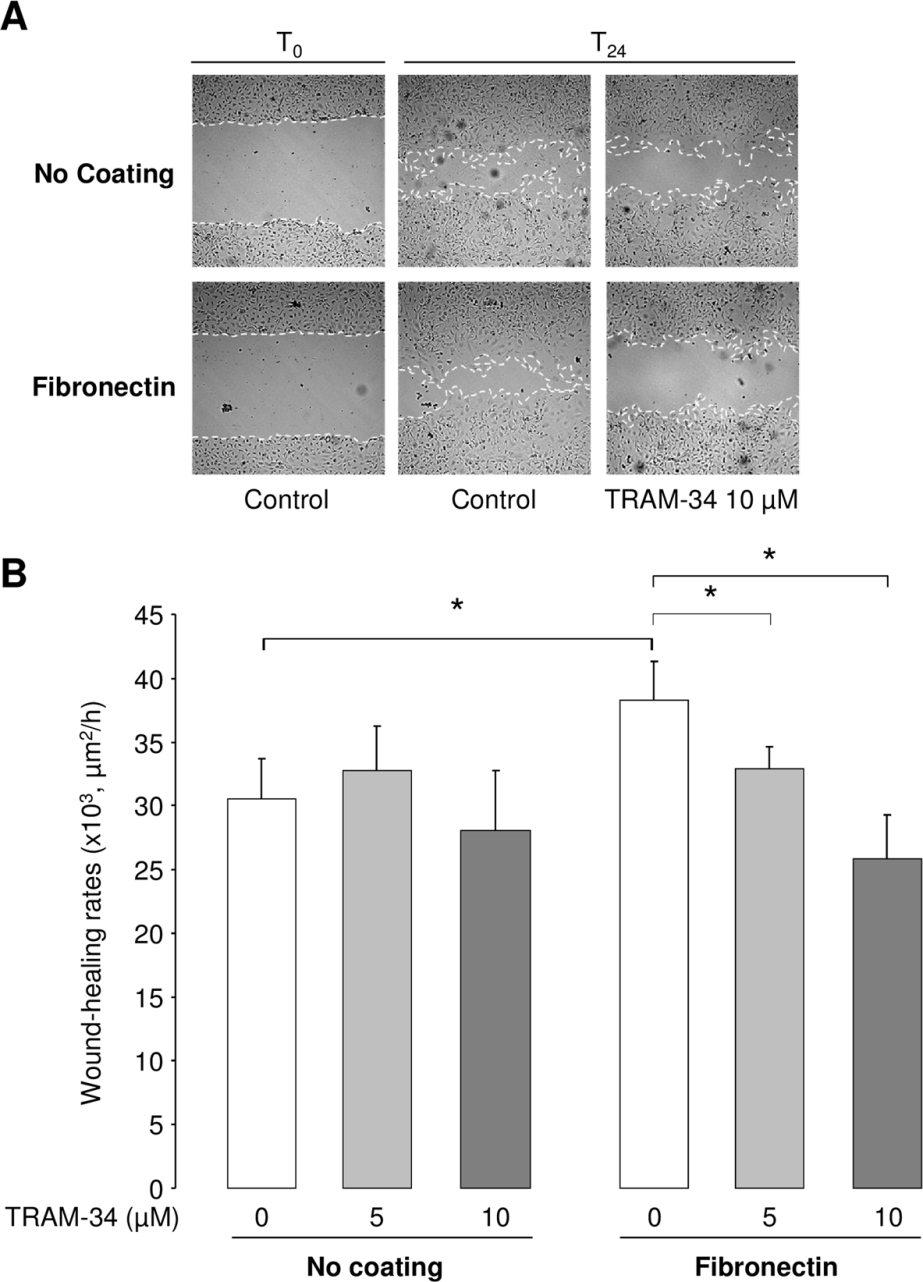
Impact of KCa3.1 inhibition on fibronectin-stimulated ATII wound repair. ATII cell monolayers, grown in the absence (no coating) or presence of fibronectin coating, were injured mechanically and wound-healing rates were measured over a 24-h period, in the presence or absence of the KCa3.1 inhibitor TRAM-34. Representative photographs (x4 magnification) at time 0 (T_0_) and 24 h (T_24_) after injury in each condition are presented in (**a**). The wound edge is indicated by the dotted lines. **b**. Mean wound-closure rates (μm^2^/h) are compared in control (0) and TRAM-34 (5 and 10 μM) treated monolayers, in the absence or presence of fibronectin coating (n = 6). *p < 0.05

A well-established wound-healing assay with mechanical injury of primary ATII cell monolayers was used to study the contribution of KCa3.1 channels in the healing process. We first observed that the presence of TRAM-34 (5 and 10 μM), a specific KCa3.1 inhibitor, did not affect the wound-healing rates of ATII cell monolayers in the absence of coating (Fig. 1b). In agreement with data from the literature [12], we then confirmed that the wound-closure rates of ATII cell monolayers grown on a fibronectin matrix were higher (38.3 ± 3.0 × 10^3^ μm^2^/h) compared to cells cultured in the absence of matrix (30.5 ± 3.1 × 10^3^ μm^2^/h), i.e. an increase of 7.8 × 10^3^ μm^2^/h (*p* < 0.04, Fig. 1a and b). This stimulatory effect was partially prevented by an anti-β1-integrin antibody (decrease of 3.5 × 10^3^ μm^2^/h, *p* < 0.02, not shown).

We also observed a dose-dependent reduction of fibronectin-stimulated wound-healing rates after KCa3.1 inhibition (15 and 33 % inhibition in the presence of 5 and 10 μM TRAM-34 respectively, Fig. 1b). In a previous study [15], we demonstrated an involvement of two other types of K^+^ channels, i.e. KvLQT1 and K_ATP_, during alveolar repair which did not required fibronectin coating. We now verified that the wound healing rate on fibronectin coating (47.0 ± 3.8 × 10^3^ μm^2^/h) was also significantly reduced in the presence of KvLQT1 and K_ATP_ inhibitors (i.e., clofilium (5 μM) and glibenclamide (10 μM), 32.1 ± 5.0 × 10^3^ μm^2^/h, *p* < 0.04, not shown). Moreover, the combination of TRAM-34 (20 μM), with clofilium and glibenclamide, elicited an additional inhibitory effect of the repair rates on fibronectin (24.4 ± 4.4 × 10^3^ μm^2^/h, *p* < 0.04, not shown).

Our data point to an involvement of KCa3.1 channels in the regulation of ATII wound repair in the presence of a fibronectin matrix. We then undertook complementary experiments to further assess the role of KCa3.1 in two crucial processes of epithelial repair, i.e. cell proliferation and migration.

### Impact of KCa3.1 inhibition on ATII cell growth

**Fig. 2:**
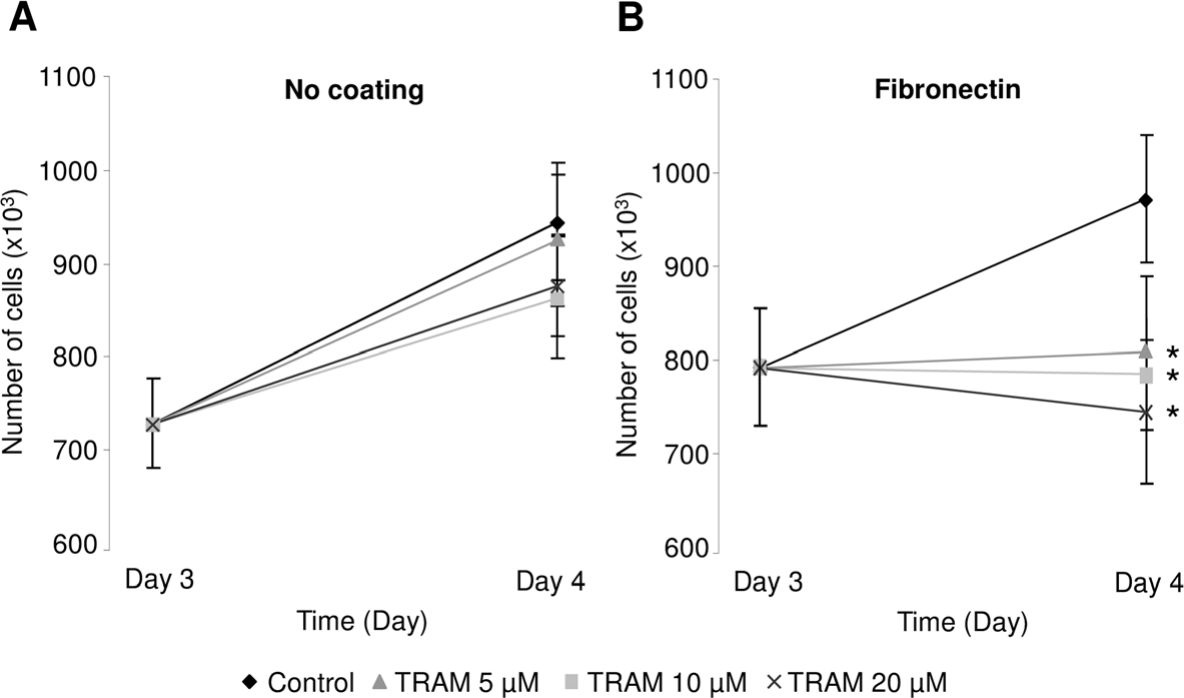
Involvement of KCa3.1 channels in ATII cell proliferation. Subconfluent ATII cells grown for 3 days in the absence (**a**) or presence of fibronectin coating (**b**) were exposed to increasing TRAM-34 concentrations (5, 10 or 20 μM) for 24 h. Cell proliferation was estimated at day 3 (T0, before treatment) and day 4 (T24h) in each condition. (n = 8). *p < 0.05

ATII cell growth was evaluated by counting the number of cells at day 3 and 4 in subconfluent primary cultures, exposed to 5, 10 and 20 μM TRAM-34 over the 24-h period (day 3 to day 4). Similar to wound-healing assays, we observed that ATII cell growth was not affected by KCa3.1 inhibition with TRAM-34 in the absence of fibronectin coating (Fig. 2a). However, TRAM-34 significantly decreased the growth of ATII cells cultured on fibronectin matrix (19, 17 and 24 % reduction in presence of 5, 10 and 20 μM TRAM-34 respectively, Fig. 2b).

### Involvement of KCa3.1 in ATII cell motility

**Fig. 3:**
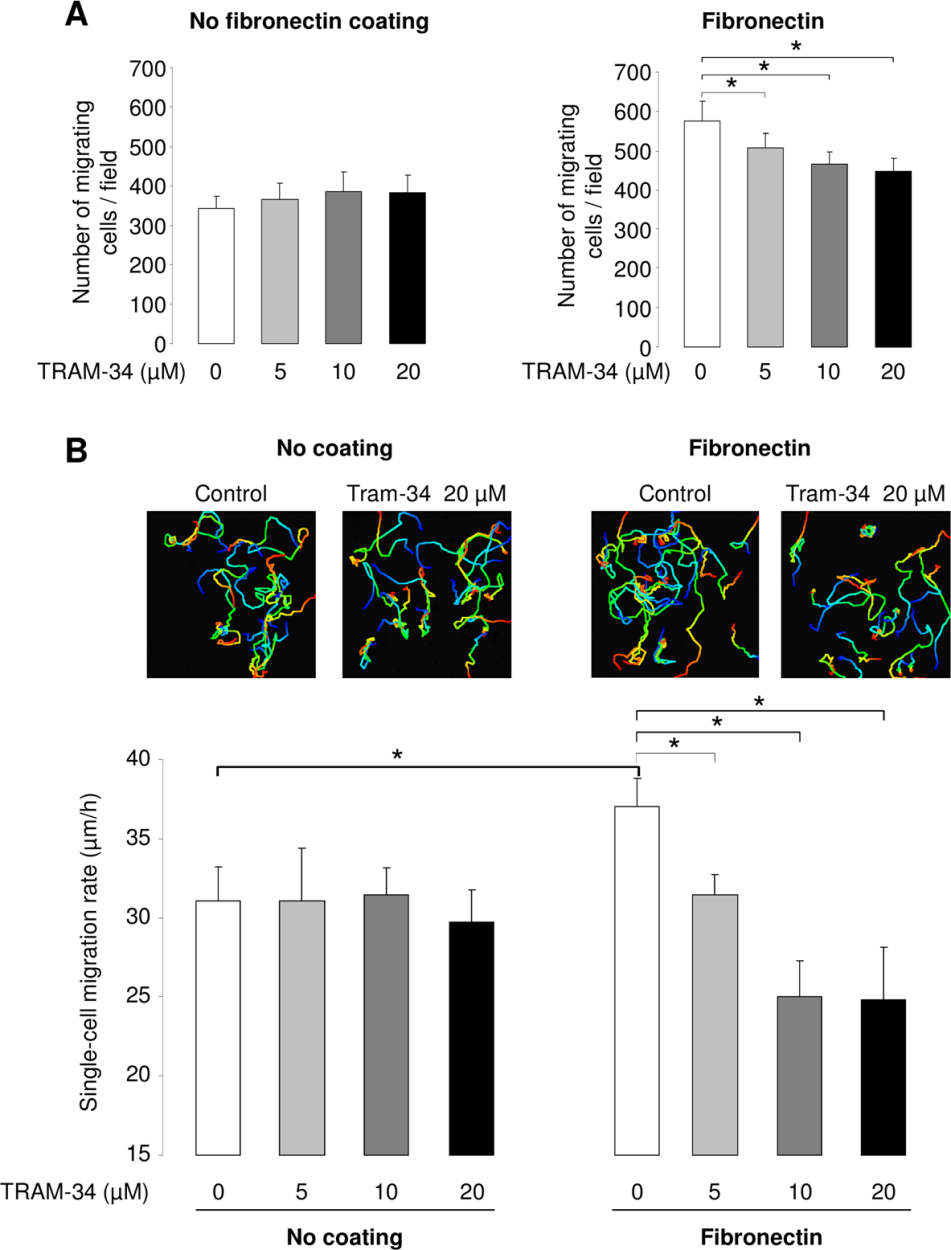
Impact of KCa3.1 inhibition on ATII cell motility. ATII cell migration was evaluated using Boyden-type assays (**a**) and single cell tracking in live time-lapse videomicroscopy (**b**). The number of migrating ATII cells were measured over an 18-h period in the absence (0) or presence of 5, 10 or 20 μM TRAM-34 and compared in the absence (**a**, no fibronectin coating, left panel) or presence of fibronectin (**a**, right panel) (5 fields/insert, 3 inserts/condition, n = 4-6). 2D cell migration rates (**b**) were evaluated by ATII single-cell tracking (~15 cells/field, 2 fields/condition/experiment, n = 6) over a 24-h period in the absence (0) or presence of TRAM-34 (5, 10 and 20 μM). Representative trajectories of ATII cells in the absence or presence of a fibronectin matrix, in control condition and in the presence of 20 μM TRAM-34 are presented in the top panel (**b**). Colors indicate 0-h (blue) to 24-h (red) time points. *p < 0.05

The role of KCa3.1 in ATII cell motility was assessed using two complementary approaches. Boyden-type chamber assays first showed that TRAM-34 exposure did not decrease the number of migrating cells in control conditions (absence of fibronectin matrix) (Fig. 3a, left panel), whereas fibronectin-stimulated cell migration was significantly reduced by TRAM-34 (12, 19 and 23 % inhibition in the presence of 5, 10 and 20 μM TRAM-34 respectively, Fig. 3a, right panel). The involvement of KCa3.1 in 2D cell migration dynamics was then assessed by single-cell tracking in subconfluent ATII cell cultures using time-lapse video-microscopy experiments. As depicted in Fig. 3b, TRAM-34 did not impact ATII cell trajectories and migration rates in the absence of coating (left panels, no coating). On the contrary, ATII cell trajectories on fibronectin matrix were altered by TRAM-34 and the mean migration rate (37 μm/h in absence of TRAM-34) was decreased to 31, 25 and 24 μm/h in the presence of 5, 10 and 20 μM TRAM-34, respectively (Fig. 3b, right panels). These results demonstrated a crucial role of KCa3.1 channels in the control of ATII cell motility on fibronectin.

### Impact of KCa3.1 silencing on wound healing, cell proliferation and migration

**Fig. 4:**
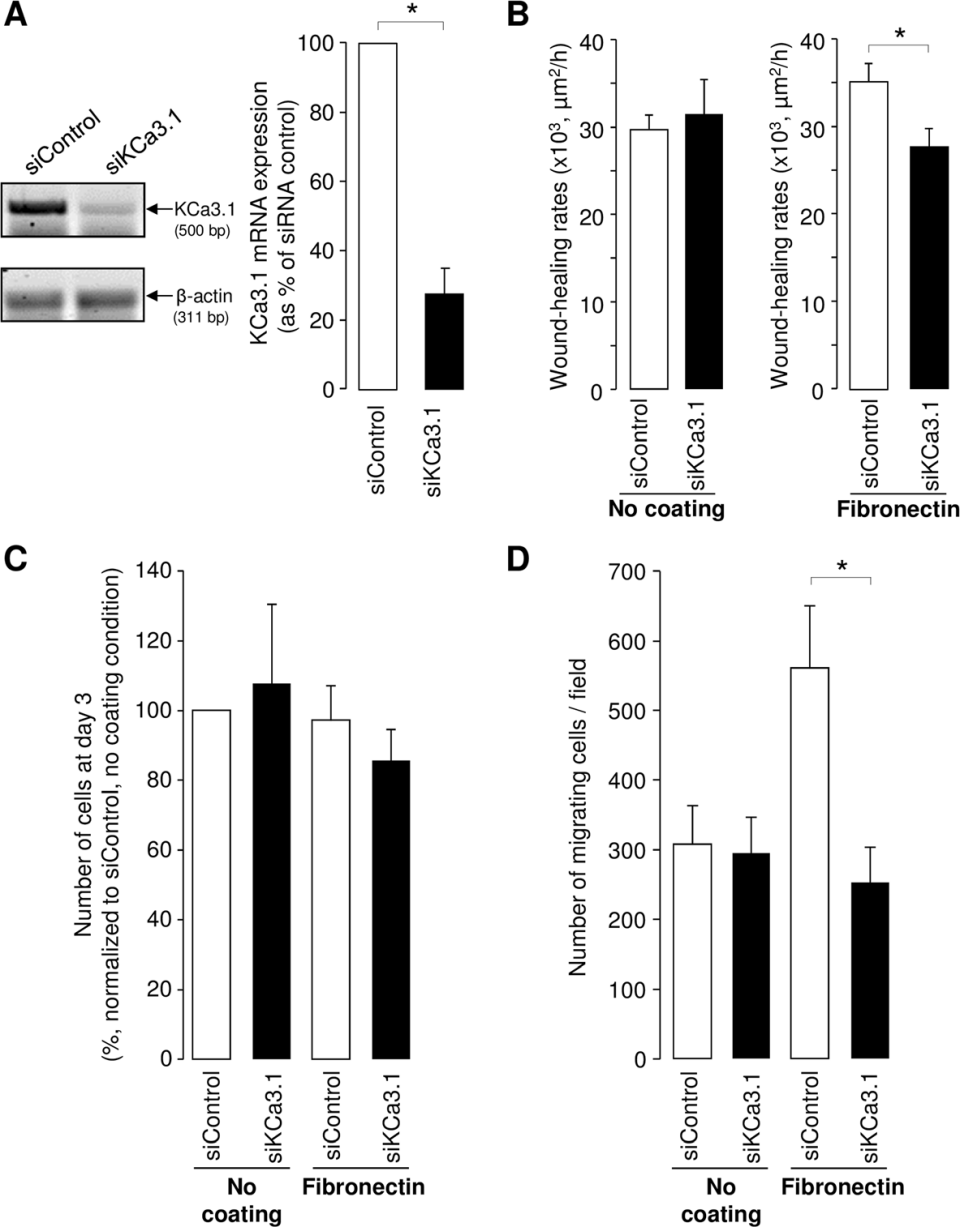
Delayed ATII wound repair and reduced cell proliferation and migration after KCa3.1 silencing in the presence of fibronectin coating. ATII cells were transfected with negative control siRNA (siRNA control) or siRNAs directed against KCa3.1 (siKCa3.1) and then seeded in the absence or presence of fibronectin coating. KCa3.1 silencing was verified by PCR (**a**, n = 7). A representative agarose gel is presented on the left. (**b**) Wound-healing rates (in μm^2^/h) were measured at 24 h after injury among cells transfected with control or KCa3.1 siRNAs in the absence of coating (left) and in the presence of fibronectin matrix (right) (n = 7). The number of ATII cells was counted at day 3 of culture after transfection with control or KCa3.1 siRNAs (n = 14, **c**). The number of migrating ATII cells, transfected 3 days before with control or KCa3.1 siRNAs, was measured in a Boyden type chamber over an 18-h period in the absence (no coating) or presence of fibronectin (n = 6, **d**). *p < 0.05

To confirm the role of KCa3.1, we then adopted a complementary approach with specific KCa3.1 siRNAs. After verification of siRNA efficiency (Fig. 4a), we showed that KCa3.1 down-regulation did not reduced alveolar repair in the absence of coating (Fig. 4b, left), whereas it was associated with a significant decrease in wound-healing (28.8 ± 2.3x10^3^ μm^2^/h in the presence of KCa3.1 siRNA), compared to ATII cells exposed to negative control siRNA (33.9 ± 1.7x10^3^ μm^2^/h, *P < 0.05,* Fig. 4b, right) on fibronectin coating. The number of ATII cells at day 3 after transfection with negative control and KCa3.1 siRNAs was similar in non-coated conditions, whereas a small, non-significant decrease in cell proliferation was noted on fibronectin coating in the presence of KCa3.1 siRNA (Fig. 4c). We also observed that the number of migrating cells through a Boyden type chamber was significantly reduced after KCa3.1 silencing (siKCa3.1) in the presence of fibronectin, but not in the absence of matrix (Fig. 4d).

### Wound healing stimulation by KCa3.1 activation

**Fig. 5:**
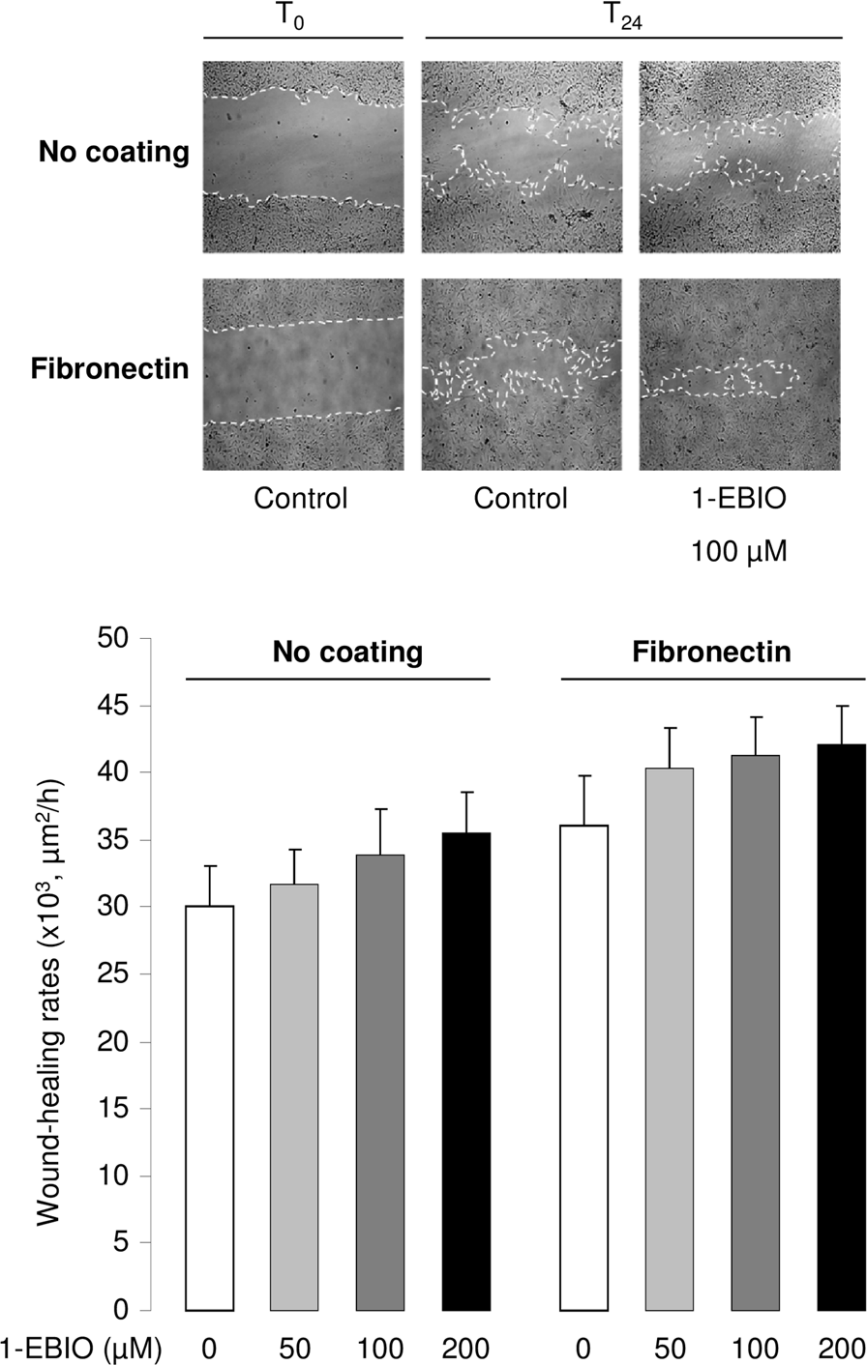
ATII wound repair stimulation with 1-EBIO. ATII cell monolayers grown for 3 days in the absence or presence of fibronectin coating were treated with increasing 1-EBIO concentrations (50, 100 and 200 μM) before wounding and wound-repair rates (in μm^2^/h) were measured over a 24-h period (n = 7). Representative photographs (x4 magnification) at time 0 (T_0_) and 24 h (T_24_) after injury in the presence or absence of 100 μM 1-EBIO are presented in the upper panel.* *p* < 0.05. Two-way Anova statistical analysis revealed that both the coating (*p* = 0.01) and 1-EBIO (*p* = 0.007) elicited a significant effect on the wound repair rates. Moreover, the values of the fibronectin group (1-EBIO (0, 50, 100, 200 μM) + fibronectin) were statistically higher than the values of the uncoated group (1-EBIO (0, 50, 100, 200 μM), no coating)

Because our data with TRAM-34 and siRNA indicated that alveolar wound repair on fibronectin matrix is, at least in part, dependent on KCa3.1 function, we next explored a potential beneficial impact of KCa3.1 activation on ATII wound-healing. As presented in Fig. 5, the repair rates of ATII cell monolayers grown in the absence of coating (30 ± 2 × 10^3^ μm^2^/h) were stimulated by increasing concentrations of 1-EBIO. This KCa activator also enhanced the fibronectin-stimulated wound repair, reaching a rate of 42 ± 3 × 10^3^ μm^2^/h in the presence of coating and 200 μM 1-EBIO.

### Relationship between KCa3.1 channels and β1-integrin

Based on our data indicating a cooperative role played by KCa3.1 and fibronectin matrix in alveolar wound repair, we then decided to further investigate a possible relationship between the KCa3.1 channel and the β1-integrin, a receptor and signal transducer of fibronectin [12].

**Fig. 6:**
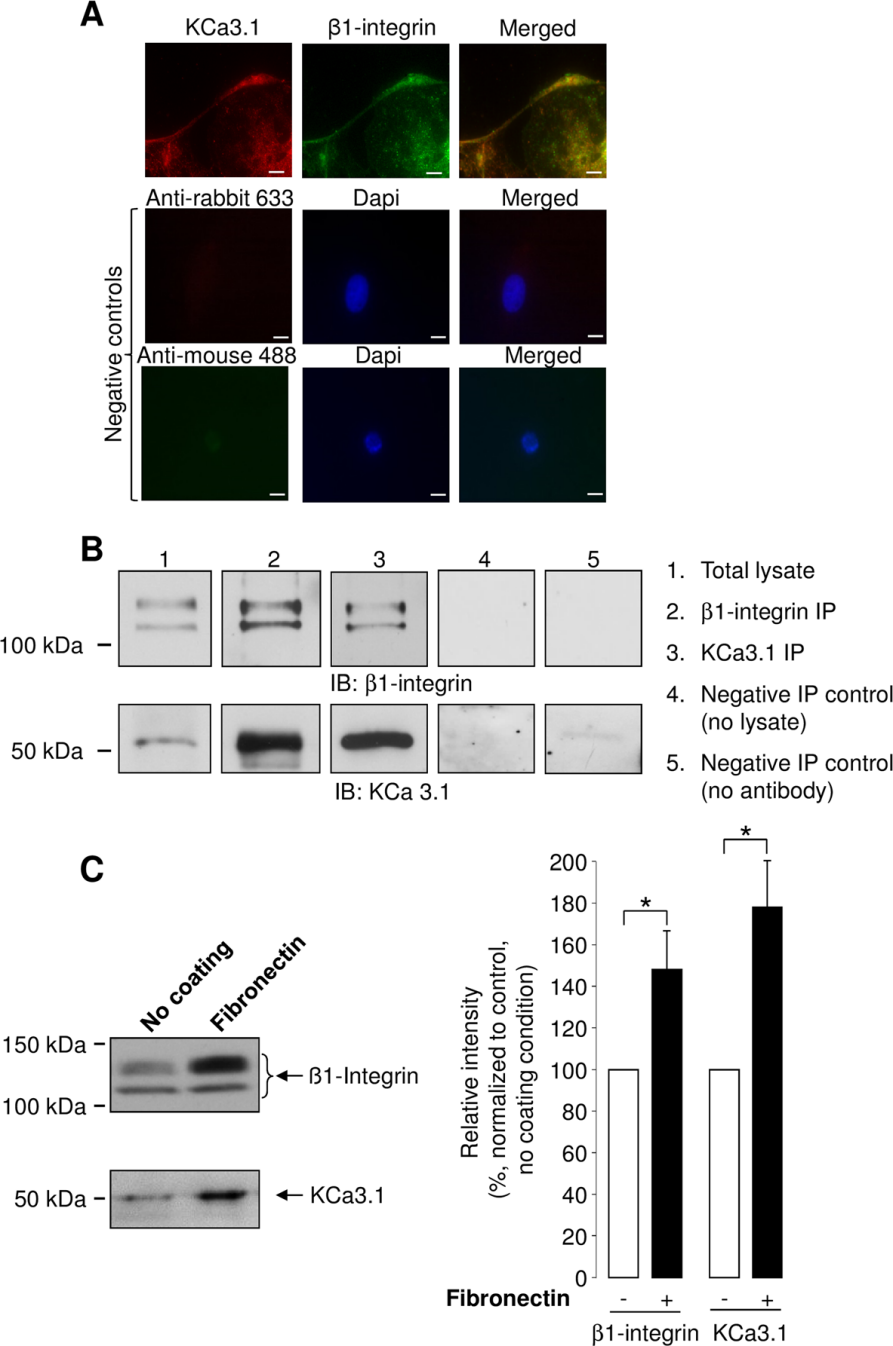
Cellular co-distribution, co-immunoprecipitation and membrane expression of β1-integrin and KCa3.1 channels. **a**. Representative immunofluorescence images of KCa3.1 and β1-integrin staining performed on ATII cells using anti-KCa3.1, anti-β1-integrin, anti-rabbit 633 (for KCa3.1 detection) and anti-mouse 488 (for β1-integrin detection) antibodies. Color superposition shows similar cellular distribution of KCa3.1 and β1-integrin in ATII cells (merge panel, Scale bars, 10 μm). No or diffuse signal was detected with the Alexa fluor 488 and Alexa fluor 633 coupled secondary antibodies in control experiments (negative controls). **b**. Representative immunoblots showing β1-integrin and KCa3.1 co-immunoprecipitations. β1-integrin (upper panels, IB: β1-integrin) and KCa3.1 (lower panels, IB: KCa3.1) proteins were revealed with specific antibodies after β1-integrin and KCa3.1 immunoprecipitation with anti-β1-integrin (lane 2 « β1-integrin IP ») or anti-KCa3.1 (lane 3 « KCa3.1 IP ») antibodies in ATII cell extracts. Endogenous expression of β1-integrin and KCa3.1 proteins in ATII cell lysate is also shown in lane 1, « Total Lysate ». Lanes 4 and 5 are negative control assays showing an absence of band in IB (IB β1-integrin and IB KCa3.1) after IP in the absence of lysate (lane 4, « Negative IP Control (no lysate) ») and in the absence of β-integrin and KCa3.1 antibodies (lane 5, « Negative IP control (no antibody) »). **c**. The level of β1-integrin and KCa3.1 channel expression in membrane fractions were determined by immunoblotting using anti-β1-integrin and anti-KCa3.1 antibodies. A representative immunoblot is shown in the left panel. The band intensities were compared in control condition (no coating, −) and in the presence of a fibronectin (+) matrix (**right panel**, n = 11). *p < 0.05

We first explored a possible spatial co-distribution of KCa3.1 channel and β-1 integrin. As shown in Fig. 6a, both KCa3.1 and β1-integrin proteins were detected by immunofluorescence assays in ATII cells. More importantly, these two proteins were predominantly co-distributed at the plasma membrane (Fig. 6a, top merge panel). It should be noted that there was no, or very diffuse, signal with secondary antibodies in control experiments in the absence of respective primary antibodies (Fig. 6a, negative controls).

We then assessed a possible assembly of these two proteins using co-immunoprecipitation assays. Expression of β1-integrin and KCa3.1 proteins was first confirmed by western blotting on ATII cell extracts (Fig. 6b, lane 1, « total lysate »), where doublets of 120–105 (mature and immature β1-integrin proteins, IB: β1-integrin) and 51 kDa (IB: KCa3.1) bands were detected using anti-β1-integrin and anti-KCa3.1 antibodies, respectively. Interestingly, KCa3.1 proteins was also detected after immunoprecipitation (IP) using anti-β1-integrin antibody (Fig. 6b, lane 2, « β1-integrin IP », IB: KCa3.1). We also detected β1-integrin proteins, after KCa3.1 immunoprecipitation (Fig. 6b, lane 3, « KCa3.1 IP », IB: β-integrin). This result thus showed for the first time that KCa3.1 could form an immunoprecipitable complex with the β-1 integrin in the ATII cells.

The expression levels of KCa3.1 and β1-integrin proteins in enriched membrane fractions of ATII cells were then compared in the presence or absence of fibronectin coating. We noted that ATII cells grown in the presence of fibronectin matrix expressed a higher level of the KCa3.1 and β1-integrin proteins in their membrane fractions (~50 % and 80 % enhancement, respectively) compared to cells cultured in the absence of coating (Fig. 6c). This observation reinforced the hypothesis of a relationship between β1-integrin and KCa3.1.

### Involvement of calcium transport in fibronectin-stimulated ATII wound repair

**Fig. 7:**
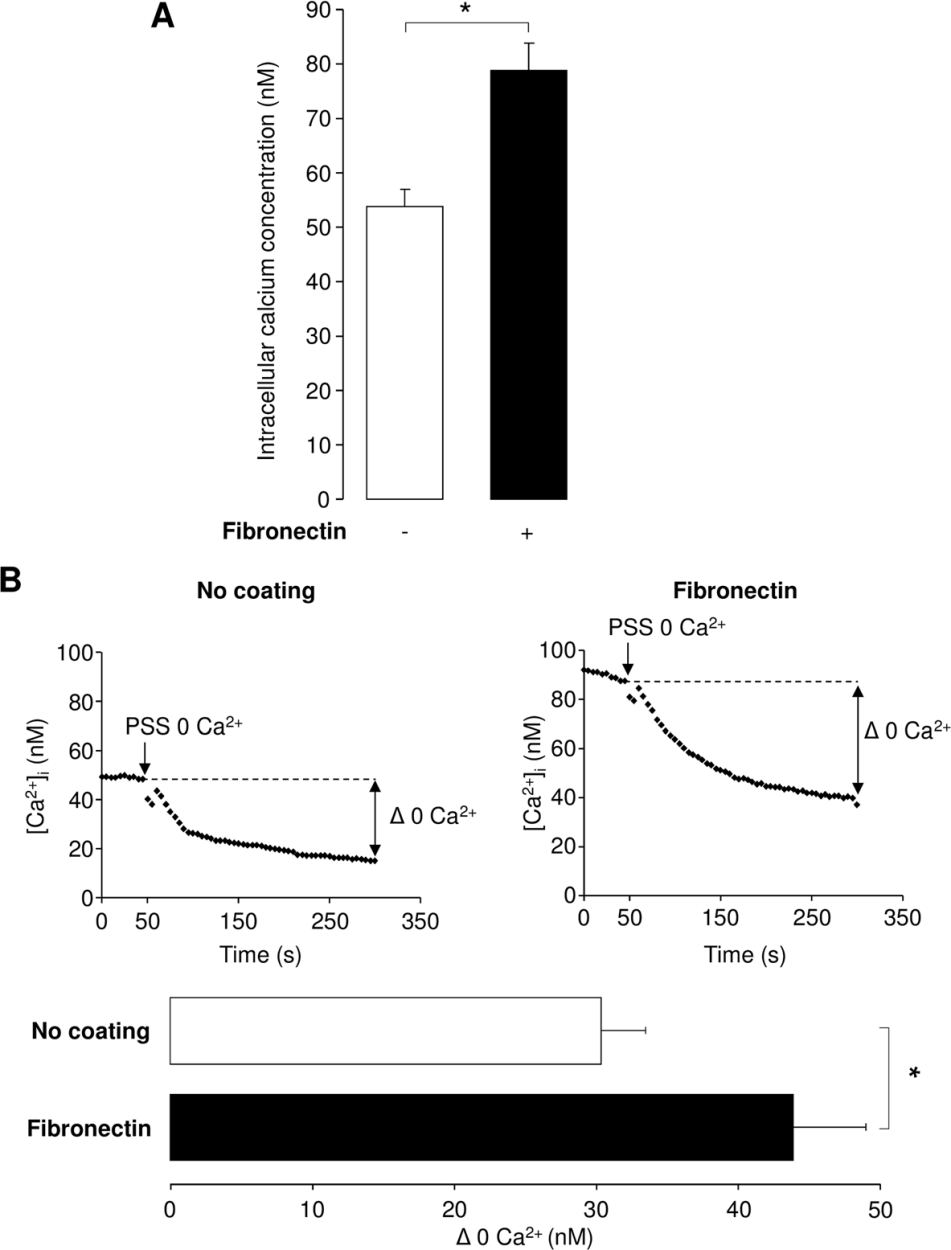
Impact of fibronectin coating on calcium signal in ATII cells. **a**. [Ca^2+^]_i_ was measured using Fura-2 in ATII cells bathed with a physiological saline solution containing 2 mM Ca^2+^ and compared in the absence (−) or presence (+) of fibronectin coating. **b.** Ca^2+^ permeability through ATII cell membranes was evaluated after external calcium removal. Illustrative traces showing the decrease of [Ca^2+^]_i_ as a function of time in absence (no coating) or in presence of fibronectin matrix (fibronectin) after PSS 0 Ca^2+^ application are presented on top panels (**b**). The mean amplitudes of [Ca^2+^]_i_ decrease were compared in the absence or in presence of fibronectin matrix (bottom panel, **b**). (n = 10) *p < 0.05

The involvement of KCa3.1, which is a calcium-activated potassium channel [46], during ATII wound repair on fibronectin, suggested that changes in intracellular calcium may occur in these conditions. We first evaluated the variation in [Ca^2+^]_i_ using Fura-2 assays in the presence and absence of fibronectin coating. We first noted that the steady-state [Ca^2+^]_i_ was higher (78.8 ± 8.8 nM) in ATII cells cultured on fibronectin compared to the control condition (absence of coating, 53.8 ± 4.3 nM, Fig. 7a). The plasma membrane Ca^2+^ permeability of ATII cells was also estimated by replacing the saline solution bathing the cells, which contained 2 mM Ca^2+^, by a Ca^2+^ free saline solution supplemented with 1 mM EGTA. This extracellular Ca^2+^ removal was followed by a reduction in [Ca^2+^]_i_ level of 30.3 ± 3.1 nM in absence of coating. Notably, the change in [Ca^2+^]_i_ was higher (43.8 ± 5.1 nM) in the presence of fibronectin (Fig. 7b).

**Fig. 8:**
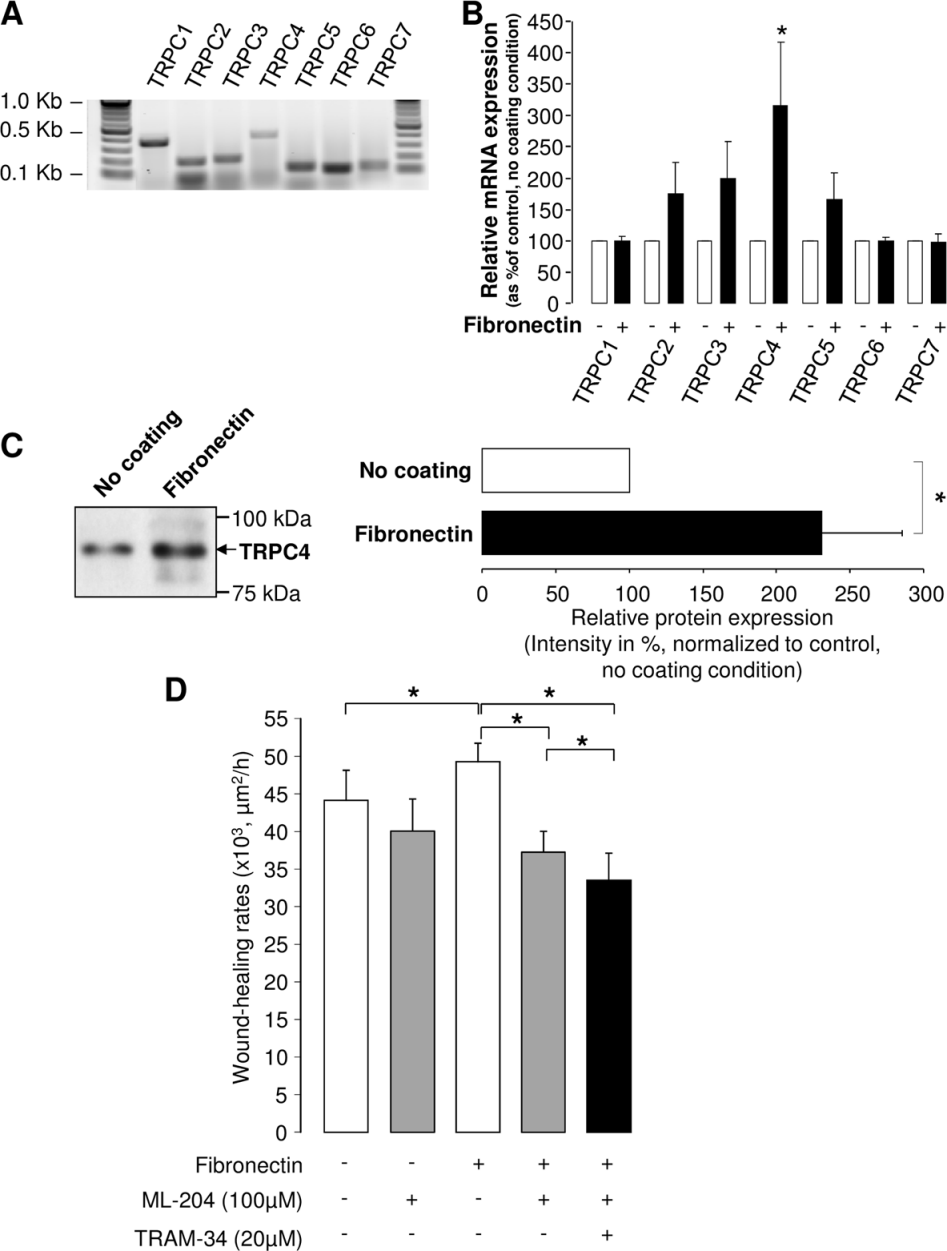
Expression of TRPC channels in ATII cells and complementary roles of TRPC4 and KCa3.1 in wound repair on fibronectin matrix. Representative agarose gel showing PCR products amplified from ATII cDNA with PCR primer pairs designed from rat TRPC channels (**a**). TRPC channel relative mRNA expressions were normalized to β-actin and compared between no coating and fibronectin matrix conditions (**b**, n = 16–20). **c**. Levels of TRPC4 protein expression in membrane fractions were measured by immunoblotting using specific anti-TRPC4 channel antibody and band intensities were compared between control (no coating) and fibronectin matrix conditions (**c**, right panel, n = 11). A representative immunoblot is shown in the left panel. **d**. ATII cells, cultured in the absence (−) or presence (+) of a fibronectin coating, were injured mechanically and the wound-healing rates (μm^2^/h) over a 24-h period were then compared in control condition (−), in presence of the TRPC4 inhibitor ML-204 (100 μM, +) (n = 8) or a combination of ML-204 (100 μM) and TRAM-34 (20 μM) (**d**, n = 7). *p < 0.05

Because calcium membrane transport mechanisms in ATII cells are not well defined, we decided to investigate the identity of Ca^2+^ channels that could be involved in the observed modifications in Ca^2+^ levels in cell grown on fibronectin. We focused on a subgroup of the large TRP channel family, the TRPC channels [47, 48], because at least one member of this subfamily has already been proposed as a functional partner of KCa3.1 [49]. Using RT-PCR, we detected the mRNA expression of all seven members of the TRPC family (TRPC1 to TRPC7, Fig. 8a). As shown in Fig. 8b, a huge enhancement in TRPC4 mRNA expression was observed in ATII cells cultured on fibronectin matrix (compared to the control, non-coated condition), whereas the increase in TRPC2, 3 and 5 mRNA was non-significant. Similar to KCa3.1 and β1-integrin proteins, we also measured an enhancement of the relative level of the TRPC4 protein expressed in the enriched membrane fraction in ATII cells cultured on fibronectin (compared to no coating, Fig. 8c).

A final series of experiments was undertaken to examine the involvement of TRPC4 channels, using the specific inhibitor ML-204 [50], in fibronectin-stimulated alveolar wound repair. As previously observed after KCa3.1 inhibition with TRAM-34 (Fig. 1b), blocking of TRPC4 channels with ML-204 (Fig. 8d, ML-204) did not significantly reduce the wound-healing rates of ATII monolayers in the absence of coating, whereas ML-204 significantly decreased the rates on fibronectin matrix (fibronectin + ML-204). Finally, an additive inhibitory effect of TRAM-34 on wound-healing rates was observed, suggesting a complementary role played by KCa3.1 and TRPC4 channels during ATII epithelial repair on fibronectin (Fig. 8d, fibronectine + ML-204 + TRAM-34).

## Discussion

**Fig. 9:**
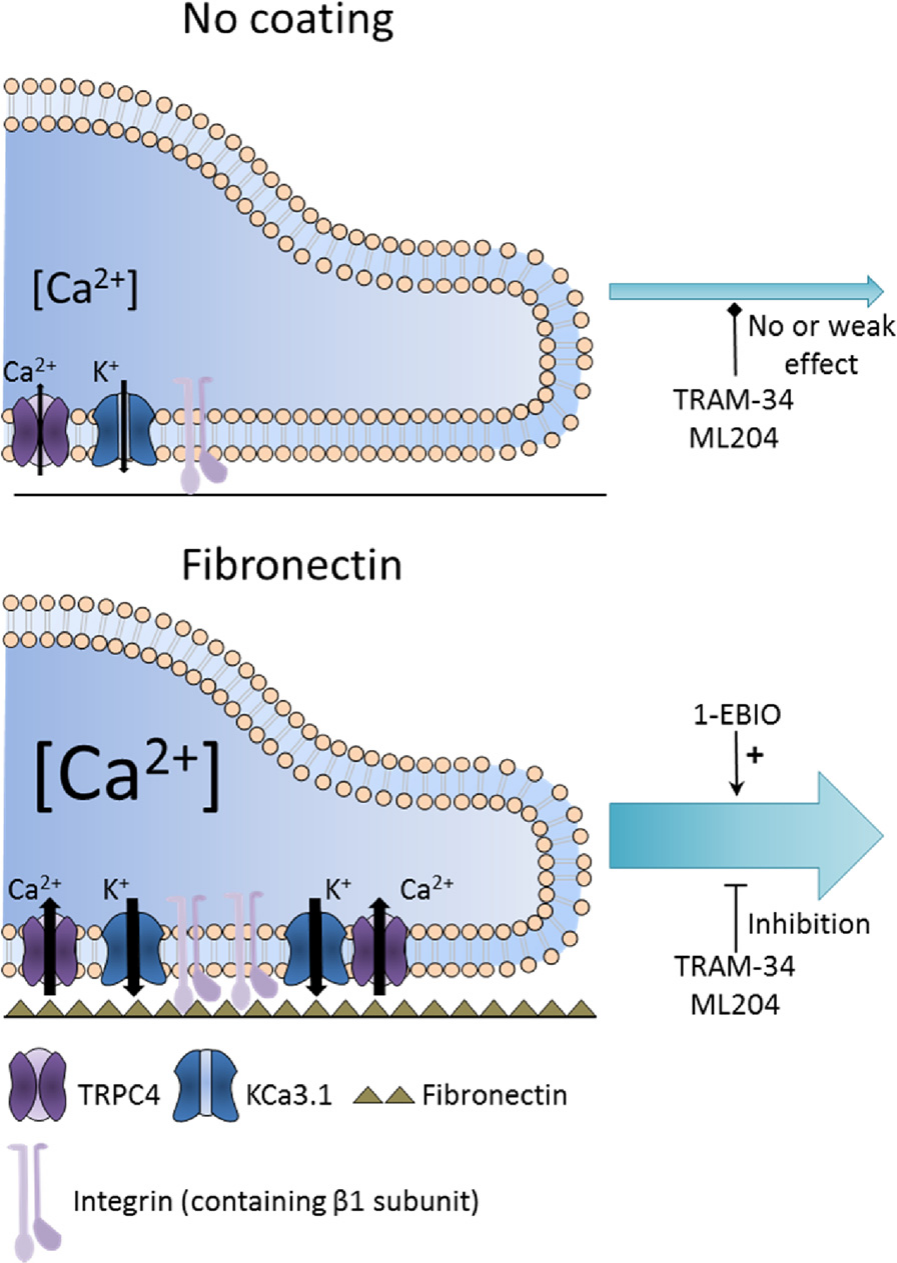
Complementary roles of ECM, β1-integrin, KCa3.1 and TRPC4 channels during alveolar repair process. The presence of fibronectin matrix evokes an increase in membrane expression of β1-integrin, KCa3.1 and TRPC4, an elevation of steady-state [Ca^2+^]_i_ and a stimulation of ATII wound healing. This stimulation is dependent, at least in part on KCa3.1 and TRPC4 channel activity. Our data also highlighted a relationship between KCa3.1 and β1-integrin

Our study revealed that Ca^2+^-activated KCa3.1 channels and interactions with integrin receptor and extracellular matrix component (fibronectin) play a significant role in alveolar wound repair processes. Specifically, we highlighted a relationship between β1-integrin, a fibronectin receptor and signal transducer, and KCa3.1 channels. We also found that the presence of a fibronectin coating is associated with elevated steady state [Ca^2+^]_i_ as well as β1-integrin, KCa3.1 and TRPC4 channel membrane expression. Finally, our data indicate complementary roles of ECM, β1-integrin, KCa3.1 and TRPC4 channels during alveolar repair processes (recapitulative scheme presented in Fig. 9).

The observed increases in ATII wound-healing (Fig. 1) and cell migration (Fig. 3) rates on fibronectin confirm importance of this extracellular matrix component in alveolar repair, in agreement with previous studies. Indeed, Kim *et al.* demonstrated using Boyden chamber assays that alveolar epithelial cell migration was increased in the presence of either soluble or coated fibronectin [12]. A role for this extracellular matrix component has also been shown in other epithelial tissues, including in the cornea [51].

One of our previous studies [15] demonstrated that KvLQT1 and K_ATP_ channels actively participate in the control of ATII wound healing in the absence of fibronectin coating, whereas our present data (Figs. 1 and 4b) indicate that KCa3.1 did not affect the wound repair rates in these conditions. Interestingly, both KCa3.1 pharmacological inhibition and molecular silencing impaired fibronectin-stimulated ATII wound-repair rates (Figs. 1 and 4b). This discrepancy could be explained by variable basal activity of KvLQT1, K_ATP_ and KCa3.1 channels in ATII cells. Indeed, it is possible that KCa3.1 channels require a signal, through β1-integrin activation in presence of fibronectin, to participate in ATII wound-healing processes, whereas KvLQT1 and K_ATP_ channels may exhibit sufficient basal activity to regulate alveolar epithelial repair even in absence of coating. In agreement with this hypothesis our data indicated an additive effect after concomitant inhibition of KvLQT1, K_ATP_ and KCa3.1 channels on fibronectin matrix. It could also be postulated that the TRAM-34-sensitivity on fibronectin coating could be due to the observed increased expression (Fig. 6c) of KCa3.1/integrin proteins, which cooperate to regulate alveolar repair, migration and proliferation in this condition. A role for KCa3.1 channels in the control of both ATII cell proliferation and migration on fibronectin (Fig. 4) was also confirmed by the siRNA approach. Such an involvement of KCa3.1 in cell motility [52–55] or proliferation [56, 57] had previously been established in other cell types.

The observation that KCa3.1 function in alveolar repair is dependent on the presence of fibronectin suggests a potential link between KCa3.1 and extracellular matrix receptors, such as integrins. In agreement with this hypothesis, our immunofluorescence and co-immunoprecipation experiments (Fig. 6) demonstrate for the first time a cellular co-distribution and a potential physical link between KCa3.1 and β1-integrin in ATII cells. Although such observations have never been reported before in native epithelial cells, physical or functional interactions between other types of K^+^ channels (e.g. BKCa, Kv1.3, hERG, K_ir_4.2) and integrins have already been reported in other cell types [30–33]. Levite *et al.*, for example, reported a physical association between Kv1.3 channels and β1-integrin, detected by co-immunoprecipitation assays, in human T cells [32]. The same approach was undertaken in a neuroblastoma cell line to identify a link between hERG1 channel and β1-integrin, which were also co-localized [30].

In the present study, we observed a significant increase in β1-integrin and KCa3.1 expression in the membrane fraction of ATII cells cultured on fibronectin coating (Fig. 6c). Although our data did not allow us to define which cellular mechanism could explain this observed up-regulation, it has been proposed in other studies that protein phosphorylation, Ca^2+^ signalling or G proteins could be responsible for the modulation of K^+^ channel properties by integrins [9, 58]. In agreement with our results, it has been also shown that the level of hERG channel co-immunoprecipitated with β1-integrin is increased after β1-integrin activation [30].

Due to the Ca^2+^ sensitivity of KCa3.1 channels, the levels of [Ca^2+^]_i_ in ATII cells in the absence or presence of a fibronectin matrix were also analyzed. Our data showed that [Ca^2+^]_i_ is significantly higher in the presence of fibronectin (Fig. 7). This result is in agreement with a study by Sjaastad *et al.,* [59] showing that integrin activation in MDCK cells, using microbeads coated with RGD motif, was associated with [Ca^2+^]_i_ increase, most probably through a Ca^2+^ influx. Our Fura-2 assays also indicated that membrane Ca^2+^ permeability in ATII cells was enhanced in the presence of fibronectin. In an attempt to define the identity of the membrane Ca^2+^ channel(s) that may be involved in that response, we decided to focus on the TRPC family, because TRPC3 channel has already been shown to facilitate the KCa3.1 channel activation [49]. Although we detected all members of the TRPC family (TRPC1-7, [47], Fig. 8) in ATII cells, only TRPC4 mRNA expression was significantly enhanced in the presence of fibronectin. Moreover, analysis of protein expression in membrane fraction revealed that TRPC4 levels, similarly to KCa3.1 and β-1 integrin, were significantly enhanced in ATII cells cultured on fibronectin coating (Fig. 8c). The signalling pathways responsible for TRPC4 up-regulation were not elucidated in our study; however, it has previously been demonstrated that TRPC4 membrane translocation was dependent of Src phosphorylation after EGF stimulation [60]; a phosphorylation pathway also known in the regulation of ion channels by integrin [9, 58]. The relative contribution of TRPC4, as well as other types of Ca^2+^ channels, including voltage-dependent and independent Ca^2+^ channels to the complex Ca^2+^ signal during wound repair or after changes in integrin and K^+^ channel activation has not been evaluated in that study but would deserve further investigation.

Finally, we observed for the first time that TRPC4 channels are involved in ATII wound healing. In fact, we measured a 3-fold stronger inhibition of ATII wound repair rates with the TRPC4 inhibitor ML204 on the fibronectin matrix than in the absence of coating. This effect is in agreement with the observed increased TRPC expression on fibronectin coating. Although a function of TRPC4 channels in alveolar repair has never been reported before, other members of the TRPC family have already been described as regulators of cell migration in non-tumoural cells (TRPC1 and TRPC3 in intestinal epithelial cells or monocytes respectively [61, 62]) or in lung cancer cells (TRPC1, 3, 4 and 6 [63]). Furthermore, we observed that the combination of KCa3.1 and TRPC4 inhibitors led to an additive inhibitory effect (Fig. 8d), suggesting that these two channels are complementary partners in the control of ATII wound healing on fibronectin.

As described above, our data using KCa3.1 inhibition or silencing demonstrated that KCa3.1 may act as a regulator of the ATII wound-healing repair *in vitro*. Interestingly, we also showed that the ATII wound-healing rates were improved in the presence of the KCa activator 1-EBIO (Fig. 5). Based on this evidence, it may be postulated that KCa3.1 could play an important role in the alveolar epithelial repair after injury and may thus be identified as a potential therapeutic target for alveolar epithelial repair after acute lung injury *in vivo*. Such a strategy remains to be further investigated, however.

## Conclusion

Our data demonstrate for the first time the complementary roles of fibronectin matrix, β-1 integrin, KCa3.1 and TRPC4 channels in the regulation of alveolar epithelial repair processes (Fig. 9). Moreover, our study highlights an improvement in ATII wound-healing after treatment with 1-EBIO, suggesting use of KCa3.1 activators as potential therapeutic strategy to favor the resolution of acute lung injury *in vivo*.
